# Cardiac parameters affect prognosis in patients with non-large atherosclerotic infarction

**DOI:** 10.1186/s10020-020-00260-5

**Published:** 2021-01-06

**Authors:** Ya-Ying Zeng, Wen-Bo Zhang, Lin Cheng, Li Wang, Dan-Dan Geng, Wen-Jie Tang, Jin-Cai He, Bin-Bin Deng

**Affiliations:** 1 Department of Neurology, The First Affiliated Hospital of Wenzhou Medical University, Wenzhou, China; 2 Department of Neurosurgery, The Children’s Hospital of Zhejiang University School of Medicine, National Clinical Research Center for Child Health, Hangzhou, China; 3 First School of Clinical Medicine, Wenzhou Medical University, Wenzhou, China; 4 Department of Gastroenterology and Hepatology, The First Affiliated Hospital of Wenzhou Medical University, Wenzhou, China

**Keywords:** Non-large atherosclerosis, Nomogram, Prognosis, Cardiac parameters

## Abstract

**Background:**

Although large artery atherosclerosis (LAA) is the most common type of cerebral infarction, non-LAA is not uncommon. The purpose of this paper is to investigate the prognosis of patients with non-LAA and to establish a corresponding nomogram.

**Patients and methods:**

Between June 2016 and June 2017, we had 1101 admissions for acute ischemic stroke (AIS). Of these, 848 were LAA and 253 were non-LAA. Patients were followed up every 3 months with a minimum of 1 year of follow-up. After excluding patients who were lost follow-up and patients who did not meet the inclusion criteria, a total of 152 non-LAA patients were included in this cohort study. After single-factor analysis and multifactor logistic regression analysis, the risk factors associated with prognosis were derived and different nomograms were developed based on these risk factors. After comparison, the best model is derived.

**Results:**

Logistics regression found that the patient’s National Institutes of Health Stroke Scale (NIHSS) score, ejection fraction (EF), creatine kinase-MB (CK-MB), age, neutrophil-to-lymphocyte ratio (NLR), aspartate aminotransferase (AST), and serum albumin were independently related to the patient’s prognosis. We thus developed three models: model 1: single NIHSS score, AUC = 0.8534; model 2, NIHSS + cardiac parameters (CK-MB, EF), AUC = 0.9325; model 3, NIHSS + CK−MB + EF + age + AST + NLR + albumin, AUC = 0.9598. We compare the three models: model 1 vs model 2, z = − 2.85, p = 0.004; model 2 vs model 3, z = − 1.58, p = 0.122. Therefore, model 2 is considered to be the accurate and convenient model.

**Conclusions:**

Predicting the prognosis of patients with non-LAA is important, and our nomogram, built on the NIHSS and cardiac parameters, can predict the prognosis accurately and provide a powerful reference for clinical decision making.

## Introduction

With the aging process, the incidence of cerebrovascular disease has been on the rise, and has become a major cause of death and disability, posing a serious threat to the life and health of the elderly (Feigin et al. 2009; Thom 2006). The problem of stroke is a huge challenge and is of great concern to scholars. Stroke sources include large artery atherosclerosis (LAA), cardioembolism, small-artery occlusion, and other causes (Adams 1993; Arsava 2017). In acute ischemic stroke (AIS), the LAA type is the most common (Deng 2019). Unfortunately, approximately 35% of ischemic events remain cryptogenic, primarily because no underlying causal mechanism is identified, even after all available diagnostic tests have been performed (Kolominsky-Rabas et al. 2001). Therefore, although LAA type occupies the majority, non-LAA still has an important position in AIS, we should still pay attention to patients with non-LAA.

Cerebral-cardiac syndrome refers to the dysfunction of the autonomic nervous system that leads to cardiovascular dysfunction after an acute encephalopathy, especially after a stroke or traumatic brain injury, often accompanied by changes in electrocardiogram and myocardial enzyme. According to Norris et al. (1979), elevated serum glutamate aminotransferase (AST), lactate dehydrogenase (LDH), and creatine kinase (CK) were observed in 8% of strokes. They also tested CK and creatine kinase-MB (CK-MB) in 230 stroke patients, 44% of whom had elevated CK and 11% of whom had elevated CK-MB. Stroke in patients with heart failure is associated with more severe neurological deficits and very high rates of cardiovascular morbidity and mortality (Hays 2006; Vemmos 2012). In addition, the association between myoglobin, aortic stiffness, CK-MB, ejection fraction (EF) and adverse outcomes in stroke patients has been widely reported (Liu et al. 2014; Rojek 2016). The clinical manifestations of cardiac damage after stroke and its impact on the patient’s prognosis have been a hot topic of discussion. Nevertheless, most research focuses on LAA type AIS, but non-LAA type is still worthy of attention, a comprehensive multifactorial predictive model can better assess the prognosis of these patients. Nomogram has been widely used to predict survival time in cancer patients. The prognostic nomogram of LAA patients has also been studied, but so far, we are not aware of any models using nomogram to assess the prognosis of non-LAA patients. The purpose of this study was to establish a nomogram to predict poor prognosis among non-LAA patients.

## Materials and methods

This study was approved by the Ethics Committee of the First Affiliated Hospital of Wenzhou Medical University and conformed to the Helsinki Declaration. Between June 2016 and June 2017, we had 1101 admissions for AIS. Of these, 848 were LAA and 253 were non-LAA. Exclusion criteria: 1, excluding other vascular infarction, cerebral venous thrombosis; 2, patients with transient ischemic attack, cerebral hemorrhage or subarachnoid hemorrhage; 3, late hospitalization (> 24 h after stroke); 4, a clear diagnosis of LAA type AIS; 5, missed follow-up or lack of outcome variables. After the inclusion and exclusion criteria, there were 152 non-LAA stroke patients were admitted to the study. In previous studies, it is common and suitable to include 2/3–4/5 patients as the development group (Gao et al. 2020; Du 2020; Wang 2020; Guan 2019). In our study, we used R (packge caret) to randomly select 2/3 of the patients as the development group, and the remaining patients as the validation group.

### Clinical and laboratory assessments

All patients underwent computed tomography immediately after admission, but the final diagnosis of AIS was confirmed by repeat computed tomography and/or magnetic resonance imaging performed between the third and seventh days after admission. At discharge, each patient received a diagnosis of the cause of the stroke. Cerebrovascular events were classified according to the TOAST criteria (Adams et al. 1998).

All blood parameters were tested in the early morning of the second day after admission, in a fasting state. Demographics, chronic diseases, hematologic parameters, and imaging findings were collected using standardized data record forms for all patients. Cardiac parameters include left ventricular diastolic diameter (LVDD), left ventricular systolic diameter (LVSD), left atrium diameter (LAD), left ventricular posterior wall thickness (LVWT), CK, CK-MB, and EF. All echocardiography was performed on the second day of admission by echocardiologists specializing in cardiac imaging. Troponin and myoglobin were not included in this study due to excessive missing data.

### Follow-up

Each patient was followed for a minimum of 1 year, and we assessed the patient’s prognosis by telephone, questionnaires, and outpatient reviews. Two independent investigators evaluated all clinical data blindly; any disagreement was resolved by a third researcher.

### Patient assessment

Patients’ National Institutes of Health Stroke Scale (NIHSS) scores were assessed on admission. Patients’ functional outcomes were assessed every 3 months by follow-up using a modified Rankin Scale (mRS) score (Banks and Marotta 2007). Good outcomes were defined as mRS scores of 0–2, while poor outcomes were defined as mRS scores of 3–6.

### Statistical analysis

Categorical variables were compared with the chi-square test, and continuous variable were compared with the nonparametric test or t-test. Logistic regression analysis was used for multivariate analysis. The nomogram was constructed via such analysis performed with rms 26 in R version. After logistic regression analysis and calculation of factors, we ranked nomogram variables using their P values (P < 0.2) and effect values and assessed the performance of the nomogram by calculating the AUC. The larger the AUC, the more accurate the prognosis is. All calculations were based on R version 3.6.1.

## Results

### Patient characteristics

General baseline data of patients is shown in Table 1. A total of 152 non-LAA patients were included in this study, with 101 randomized as the development group and 51 as the validation group. In the development group, the mean age of patients with good outcome (mRS ≤ 2) was 65.64 ± 13.61, with 59.4% male predominance; whereas the mean age of patients with poor outcome (mRS > 2) was 73.97 ± 9.55, with 71.9% male predominance. Table 2 shows that cardiac parameters have no effect on the prognosis of LAA-type AIS patients.

**Table 1:** Demographic and clinical characteristics

	Total			Development cohort		
	Good	Poor	P	Good	Poor	P
Male	57(59.4%)	37(66.1%)	0.49	41(59.4%)	23(71.9%)	0.271
Age	66.46 ± 13.33	74.71 ± 9.06	< 0.001*	65.64 ± 13.61	73.97 ± 9.55	0.002*
DM	25(26.0%)	18(32.1%)	0.458	21(30.4%)	11(34.4%)	0.819
Smoking	37(38.5%)	19(33.9%)	0.605	24(34.8%)	10(31.3%)	0.823
Drinking	34(35.4%)	15(26.8%)	0.287	20(29.0%)	7(21.9%)	0.629
SBP	148.73 ± 22.40	148.55 ± 19.86	0.96	153.26 ± 21.65	150.00 ± 22.49	0.496
DBP	80.68 ± 13.55	82.09 ± 14.51	0.554	82.74 ± 13.10	81.78 ± 13.98	0.745
NIHSS	2.69 ± 1.98	8.75 ± 5.92	< 0.001*	2.52 ± 1.88	8.50 ± 6.55	< 0.001*
Neutrophils	4.18 ± 1.80	5.52 ± 2.42	< 0.001*	4.28 ± 1.80	5.66 ± 2.49	0.005*
Lymphocyte	1.71 ± 0.75	1.29 ± 0.43	< 0.001*	1.76 ± 0.71	1.32 ± 0.53	< 0.001*
NLR	3.44 ± 4.34	4.72 ± 2.96	< 0.001*	3.39 ± 4.68	5.66 ± 2.49	< 0.001*
AST	26.07 ± 10.88	30.93 ± 11.20	0.006*	25.16 ± 11.30	31.00 ± 11.52	0.008*
ALT	23.80 ± 16.66	23.33 ± 19.03	0.377	23.61 ± 17.72	27.07 ± 23.40	0.917
TB	12.71 ± 8.56	12.69 ± 6.66	0.608	11.86 ± 7.22	12.19 ± 7.08	0.970
DB	4.84 ± 2.94	4.99 ± 2.18	0.568	4.49 ± 2.28	4.55 ± 2.06	0.286
IB	8.34 ± 6.01	7.77 ± 4.41	0.193	8.03 ± 5.38	7.52 ± 4.55	0.686
RBC	4.88 ± 4.14	4.30 ± 0.61	0.189	5.04 ± 4.89	4.26 ± 0.59	0.199
PLT	215.77 ± 60.59	227.07 ± 79.96	0.363	224.94 ± 63.88	235.31 ± 67.38	0.468
HB	134.84 ± 18.47	128.88 ± 18.71	0.061	134.24 ± 17.99	126.16 ± 15.70	0.032*
Cr	80.26 ± 55.07	78.75 ± 29.07	0.825	81.77 ± 63.56	83.19 ± 35.34	0.886
Bun	5.71 ± 2.70	9.83 ± 24.41	0.103	5.59 ± 2.88	13.31 ± 32.29	0.050*
TC	4.64 ± 1.11	4.09 ± 1.14	0.006*	4.79 ± 1.18	4.21 ± 0.96	0.013*
TG	1.69 ± 1.11	1.29 ± 0.58	0.016*	1.73 ± 1.13	1.28 ± 0.50	0.042*
HDL	1.16 ± 0.32	1.06 ± 0.29	0.044*	1.16 ± 0.32	1.03 ± 0.24	0.031*
LDL	2.54 ± 0.76	2.32 ± 0.85	0.125	2.63 ± 0.80	2.46 ± 0.79	0.363
CK	111.60 ± 125.42	209.33 ± 347.08	0.022*	91.64 ± 59.71	241.25 ± 429.51	0.01*
CK-MB	13.59 ± 5.48	19.16 ± 8.01	< 0.001*	13.69 ± 5.40	20.16 ± 8.77	< 0.001*
Na	140.34 ± 2.64	139.53 ± 3.19	0.115	140.26 ± 2.53	139.94 ± 2.47	0.548
K	3.86 ± 0.32	3.93 ± 0.41	0.261	3.90 ± 0.31	3.96 ± 0.43	0.474
Hb1C	6.37 ± 1.26	5.94 ± 0.68	0.193	6.49 ± 1.38	6.02 ± 0.69	0.139
T3	1.18 ± 0.30	1.02 ± 0.39	0.008*	1.17 ± 0.28	0.97 ± 0.28	0.001*
TSH	2.74 ± 6.10	2.25 ± 2.08	0.483	2.53 ± 6.87	1.89 ± 1.56	0.467
LVDD	47.99 ± 6.77	49.02 ± 6.54	0.384	49.09 ± 4.76	47.41 ± 6.88	0.359
LVSD	31.73 ± 4.82	33.55 ± 6.89	0.072	32.91 ± 4.47	32.75 ± 6.88	0.926
LAD	43.25 ± 6.88	45.92 ± 7.39	0.041*	42.63 ± 6.64	45.41 ± 8.04	0.111
IST	10.65 ± 1.72	11.08 ± 2.27	0.248	10.79 ± 1.81	11.31 ± 2.56	0.331
LVWT	10.33 ± 1.24	10.55 ± 1.17	0.302	10.37 ± 1.09	10.41 ± 1.12	0.864
EF	64.68 ± 6.62	58.32 ± 10.16	< 0.001*	66.20 ± 5.53	58.10 ± 11.00	< 0.001*
Albumin	38.12 ± 3.19	34.86 ± 3.79	< 0.001*	38.14 ± 3.09	34.42 ± 3.53	< 0.001*

*DM* diabetes mellitus, *SBP* systolic blood pressure, *DBP* Diastolic blood pressure, *NLR* neutrophil-to-lymphocyte ratio, *ALT* alanine aminotransferase, *AST* aspartate aminotransferase, *TB* total bilirubin, *DB* direct bilirubin, IB indirect bilirubin, *RBC* red blood cell, *PLT* platelet, *HB* hemoglobin, *Cr* creatinine, *Bun* blood urea nitrogen, *TC* total cholesterol, *TG* triglyceride, *HDL* high density lipoprotein, *LDL* low density lipoprotein, *CK* creatine kinase, *CK-MB* creatine kinase-MB, *T3* triiodothyronine, *TSH* thyroid stimulating hormone, *LVDD* left ventricular diastolic diameter, *LVSD* left ventricular systolic diameter, *LAD* left atrial diameter, *LVWT* left ventricular septal thickness, *EF* ejection fraction, *NIHSS*: National Institutes of Health Stroke Scale on admission, *p < 0.05

**Table 2:** Cardiogenic parameters of LAA and non-LAA type stroke

	Non-LAA			LAA		
	Good	Poor	P	Good	Poor	P
CK	111.60 ± 125.42	209.33 ± 347.08	0.022*	99.37 ± 121.58	126.05 ± 181.61	0.839
CK-MB	13.59 ± 5.48	19.16 ± 8.01	< 0.001*	17.39 ± 23.47	14.32 ± 6.54	0.353
LVDD	47.99 ± 6.77	49.02 ± 6.54	0.384	48.53 ± 8.20	47.54 ± 5.17	0.206
LVSD	31.73 ± 4.82	33.55 ± 6.89	0.072	31.64 ± 16.18	30.54 ± 4.74	0.273
LAD	43.25 ± 6.88	45.92 ± 7.39	0.041*	39.88 ± 4.72	39.23 ± 4.66	0.213
LVWT	10.33 ± 1.24	10.55 ± 1.17	0.302	10.70 ± 1.36	10.80 ± 1.23	0.660
EF	64.68 ± 6.62	58.32 ± 10.16	< 0.001*	64.64 ± 9.86	65.57 ± 6.16	0.903

*CK* creatine kinase, *CK-MB* creatine kinase-MB, *LVDD* left ventricular diastolic diameter; *LVSD* left ventricular systolic diameter; *LAD* left atrial diameter, *LVWT* left ventricular septal thickness, *EF* ejection fraction, *p < 0.05

Univariate analysis found that the patient’s age, NIHSS on admission, lymphocytes, neutrophils, neutrophil-to-lymphocyte ratio (NLR), hemoglobin (HB), blood urea nitrogen (Bun), total cholesterol (TC), triglyceride (TG), high density lipoprotein (HDL), creatine kinase (CK), creatine kinase-MB (CK-MB), triiodothyronine (T3), ejection fraction (EF), albumin, these factors were statistically different. Univariate and multivariate analysis were used to identify potential prognostic factors in non LAA patients, we used logistic regression to this end. We consider the age of the patient (OR: 1.134, 95% CI: 0.963–1.336), NIHSS (OR: 2.005, 95% CI: 1.242–3.234), NLR (OR: 1.569, 95% CI: 0.925–2.663), albumin (OR: 0.554, 95% CI: 0.255–1.203), CK-MB (OR: 1.126, 95% CI: 0.951–1.333), EF (OR: 0.696, 95% CI: 0.470–1.030), AST (OR: 0.858, 95% CI: 0.684–1.076), a total of 7 variables have independent effects on the prognosis of patients (P < 0.2) (Table 3).

**Table 3:** Multivariate logistic regression according to the functional outcomes

	OR	95%CI	P
Age	1.134	0.963–1.336	0.132^#^
NIHSS	2.005	1.242–3.234	0.004^#^
NLR	1.569	0.925–2.663	0.095^#^
Albumin	0.554	0.255–1.203	0.136^#^
TC	0.423	0.111–1.605	0.206
TG	3.021	0.515–17.713	0.220
HDL	0.096	0.000–19.366	0.382
CK	1.003	0.996–1.010	0.434
CK-MB	1.126	0.951–1.333	0.170^#^
T3	3.632	0.036–368.991	0.584
EF	0.696	0.470–1.030	0.070^#^
AST	0.858	0.684–1.076	0.185^#^
BUN	1.433	0.595–3.449	0.422

*NLR* neutrophil-to-lymphocyte ratio, *AST* aspartate aminotransferase, *Bun* blood urea nitrogen, *TC* total cholesterol, *TG* triglyceride, *HDL* high density lipoprotein, *CK* creatine kinase, *CK-MB* creatine kinase-MB, *T3* triiodothyronine, *EF* ejection fraction, *NIHSS* National Institutes of Health Stroke Scale on admission, ^#^p < 0.2

**Fig. 1:**
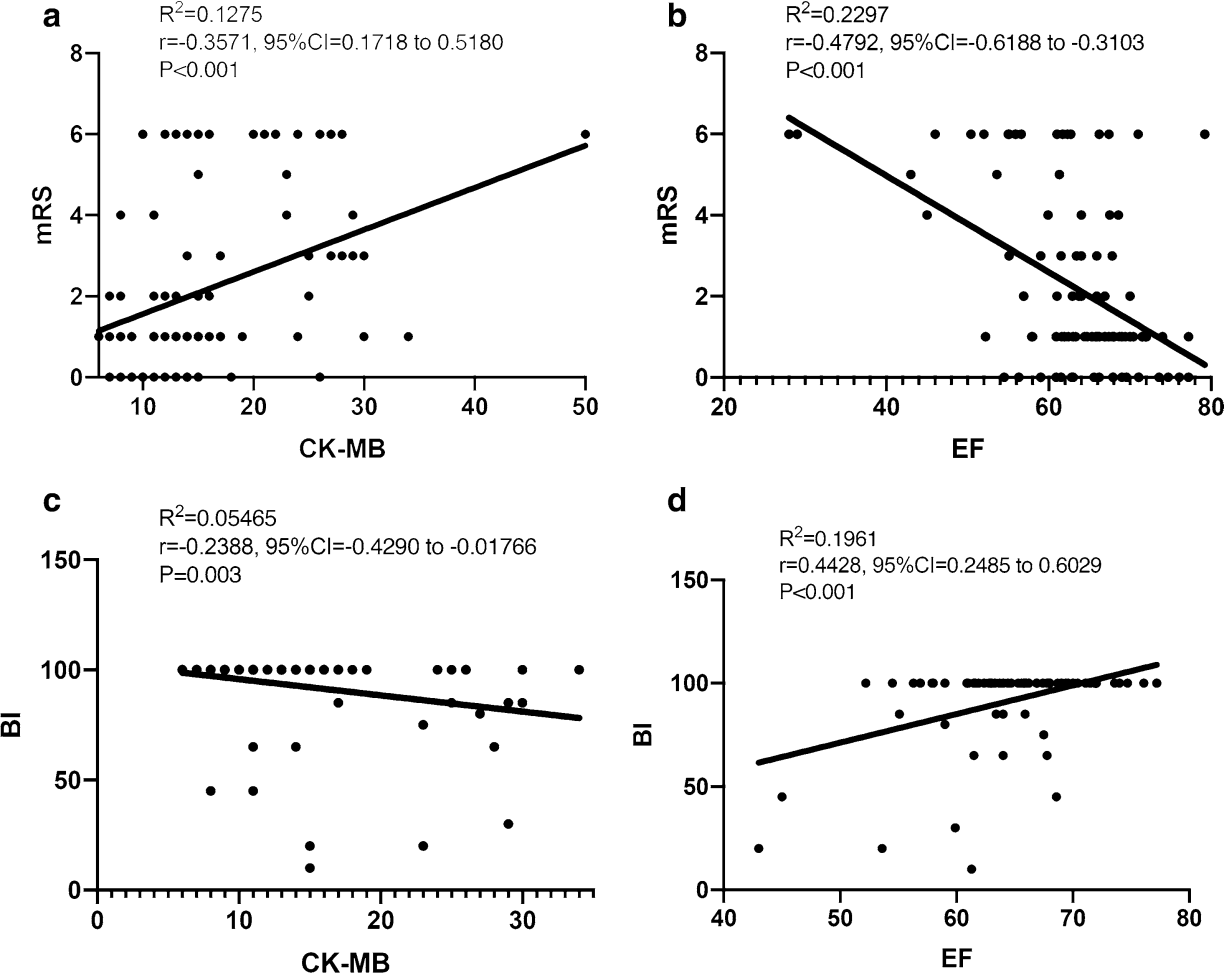
Correlation between cardiac parameters and prognosis of non-LAA patients. *Non-LAA* non-large artery atherosclerosis

To investigate whether cardiac parameters were independently associated with a patient’s prognosis (mRS, BI), we performed a Pearson correlation analysis. As shown in Fig. 1, patients’ CK-MB and mRS, CK-MB and BI, EF and mRS, EF and BI were all independently correlated.

**Fig. 2:**
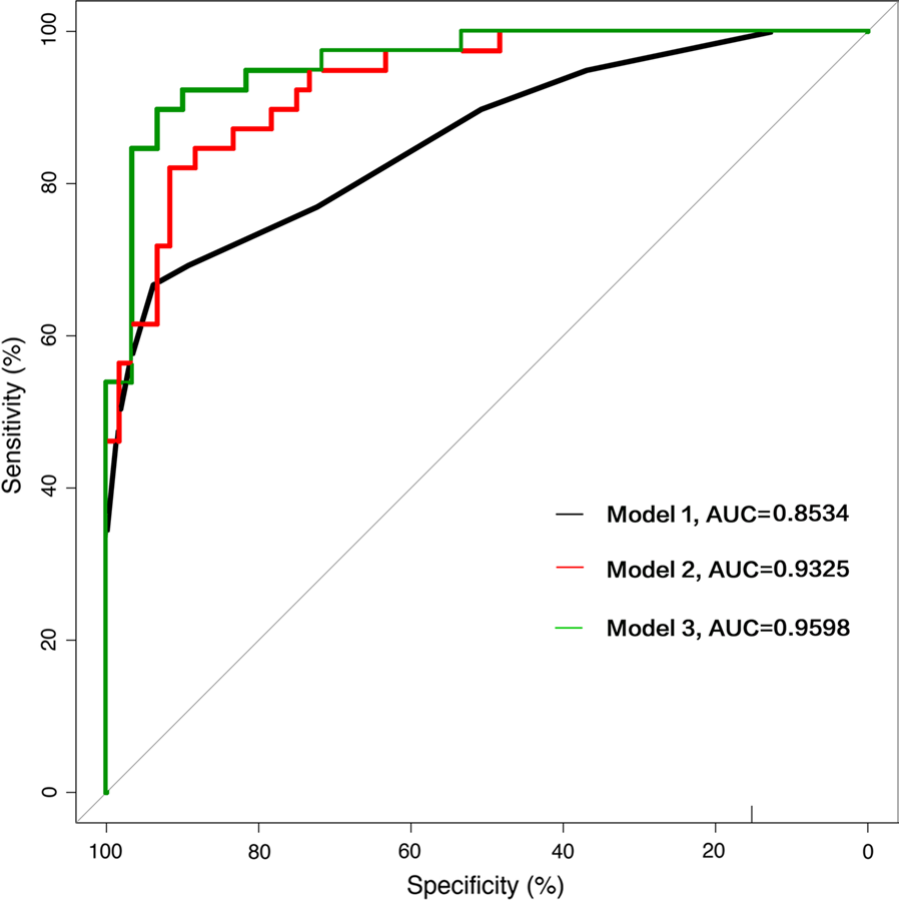
The ROC curves of the three models. Model 1: single NIHSS score, AUC = 0.8534; Model 2, NIHSS + cardiac parameters (CK-MB, EF), AUC = 0.9325; Model 3, NIHSS + CK-MB + EF + age + AST + NLR + albumin, AUC = 0.9598. We compare the three models: model 1 vs model 2, z = − 2.85, p = 0.004; model 2 vs model 3, z = − 1.58, p = 0.122

**Fig. 3:**
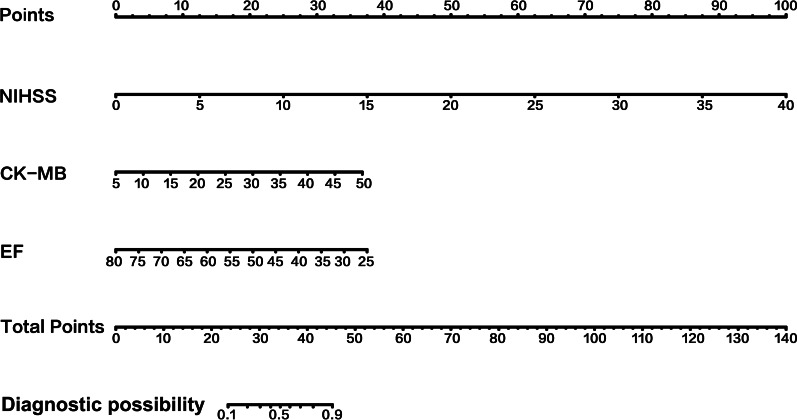
The nomogram for patients with non-LAA-type AIS. To use the nomogram, an individual patient’s value is located on each variable axis, and a line is drawn upward to determine the number of points received for each variable value. The sum of these numbers is located on the Total Points axis, and a line is drawn downward to the survival axes to determine the likelihood of poor outcome

On this basis, we have constructed three models, through comparison, we will select the best model to build nomogram. Model 1: single NIHSS score, AUC = 0.8534; Model 2, NIHSS + cardiac parameters (CK-MB, EF), AUC = 0.9325; Model 3, NIHSS + CK-MB + EF + age + AST + NLR + albumin, AUC = 0.9598. We compared the three models: model 1 vs model 2, z = − 2.85, p = 0.004; model 2 vs model 3, z = − 1.58, P = 0.122 (Fig. 2). As shown above, on the basis of model 1, the accuracy of the model is significantly improved after the inclusion of cardiac parameters. Compared with model 2, the AUC of model 3 has increased from 0.9325 to 0.9598. However, this is a model based on the additional inclusion of four other variables, which are far more complex to assess clinically than model 2. In addition, no significant difference was observed between the two models, P = 0.122. Clearly, model 2 is the optimal solution for assessing the prognosis of non-LAA patients, and the resulting nomogram is shown in Fig. 3.

**Fig. 4:**
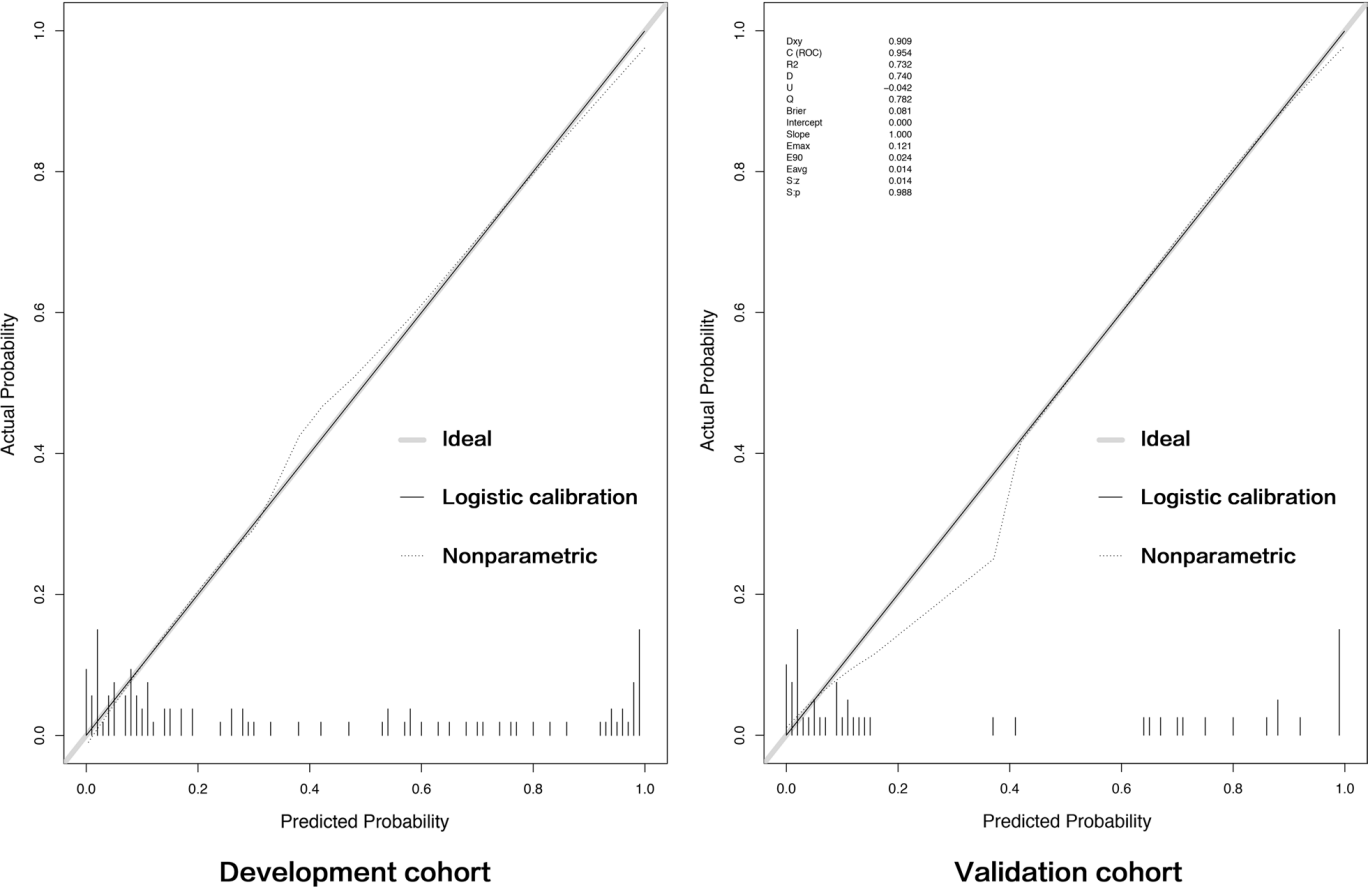
Calibration plot of nomogram

**Fig. 5:**
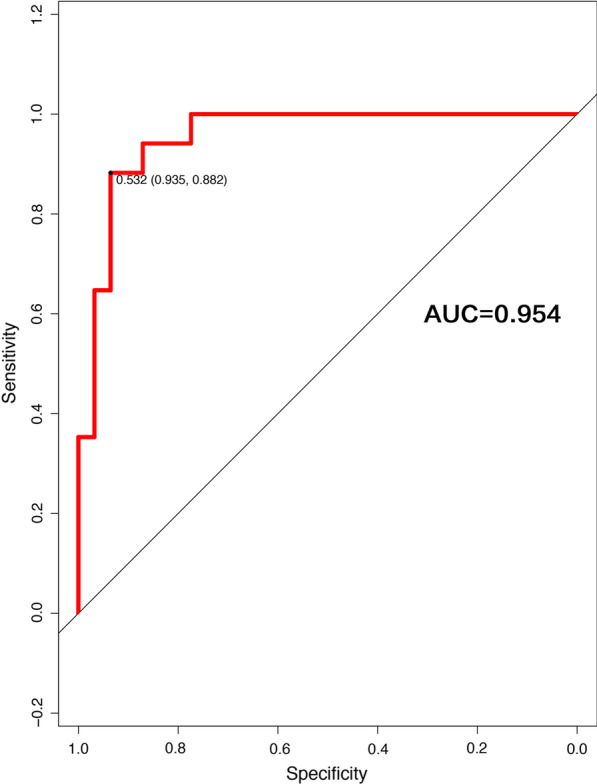
ROC curve of validation group. *Non-LAA* non-large artery atherosclerosis, *AIS* acute ischemic stroke, *CK-MB* creatine kinase-MB

The prognostic calibration plot after one year of follow-up in non-LAA patients showed the best agreement between nomogram predictions and actual observations. In both the modeling and validation groups, the calibration plot matched the actual conditions almost perfectly (Fig. 4). In the validation group, we can see that the area under the curve of the ROC reaches 0.954, further illustrating the excellent predictive ability of the model (Fig. 5).

## Discussion

Using a development cohort derived from the prospective continuous hospital stroke registry of the First Affiliated Hospital of Wenzhou Medical University, we have established a nomogram with high predictive ability. We found that the model based on NIHSS scores plus cardiac parameters balanced accuracy and simplicity.

LAA is a key subtype of the Trial of Org 10,172 in Acute Stroke Treatment (TOAST) classification system, accounted for 50.74% of all AIS in the work of Deng (2019). It was also the most common AIS subtype in our research. Non-LAA usually includes cardioembolism, small-artery occlusion, and other causes. Most studies have focused on LAA patients, while few studies have looked at non-LAA patients. Deng et al. completed the nomogram of LAA type AIS in the research (Deng 2019). In their nomogram, diabetes, NIHSS, and TG/HDL were included as risk factors for the prognosis of LAA patients. In the nomogram we established previously, we found that age, systolic blood pressure, NIHSS, and neutrophil-to-lymphocyte ratio of LAA patients were risk factors that affect the prognosis. However, in this article, the prognosis of patients with non-LAA AIS is related to NIHSS and cardiogenic parameters. This may be due to cardiac parameters caused a considerable number of patients with cerebral infarction. Whether to include cardiac parameters is the biggest difference between LAA and non-LAA nomogram.

Changes in cardiac parameters caused by stroke have been widely reported. The mechanism of cardiac alterations secondary to acute cerebrovascular disease may be: (1) Imbalance of cardiac autonomic nervous regulation. Hypothalamus, brain stem and limbic system are the regulatory centers of the cardiac autonomic nerve, which regulate the activities of sympathetic and parasympathetic nerves. Injury to these areas can cause uncontrolled regulation of sympathetic and parasympathetic balance, resulting in dysfunctional regulation of the cardiovascular nerve center (2006); (2) Disturbance of neurohumoral regulation. Various craniocerebral diseases cause damage to the nerve center and release large amounts of transmitters such as catecholamine into the bloodstream. It has been found that the degree of brain damage is directly proportional to the release of catecholamines, and the more severe the neurocentral damage, the higher the concentration of catecholamines in the peripheral blood. High concentrations of catecholamines in the blood cause platelet aggregation and thrombus formation, blocking the small blood vessels of the heart and causing local myocardial ischemia (Soblosky 1992). Significantly elevated catecholamines can cause coronary artery spasm and even necrotic changes in the myocardium, resulting in an increased myocardial enzyme (Wei et al. 2002; Orihara 2000); (3) Electrolyte disorders. When the occurrence of acute cerebrovascular, often appear electrolyte disorders, such as low potassium, low sodium, low chlorine. If the thalamus is damaged, it will lead to neuromodulation disorder, causing potassium in the extracellular fluid to be transferred into the muscle and liver cells, resulting in disturbance of potassium metabolism, hypokalemia and damage to the heart (Singh et al. 2002; Fabinyi et al. 1977).

Although myocardial damage can occur after a stroke, it is still unclear how stroke regulates heart function, what is the direct effect of stroke on heart function, and what is the underlying molecular mechanism. In a review, Chen et al. summarized the mechanism of brain–heart interaction (Chen 2017a): Hypothalamic–pituitary–adrenal (HPA) axis, catecholamine surge and sympathetic and parasympathetic regulation after stroke; Blood brain barrier disruption after stroke; Immunoresponse and systemic inflammation after stroke; Gut microbiome dysbiosis after stroke. MicroRNA also plays an important role in brain–heart interaction (Han et al. 2015; Min 2009). Chen et al. (2017b) found in the study that compared with non-stroke animals, stroke mice have significantly lower heart EF, meanwhile, atrogin-1 and E3 ubiquitin ligase murf-1 were elevated, and the transcription factor peroxisome proliferative activated receptor was a potential mediator of transcriptional dysregulation in stroke-related myocardial atrophy (Veltkamp 2019). At the same time, stroke significantly increased macrophage infiltration and increased levels of Interleukin-1(IL-1), Interleukin-6(IL-6), Monocyte-chemoattractant protein-1(MCP-1), tumor necrosis factor-β(TGF-β) and macrophage-related inflammatory cytokines in the heart, and induced cardiac fibrosis (Yan 2020). And cell death, including increased positive expression of tumor necrosis factor-α (TNF-α), Caspase 3, Microtubule Associated Protein 1 Light Chain 3 Alpha (MAP1LC3A) in the heart, occurred in chronic ischemic stroke mice (Ishikawa 2013). MiR-126 deficiency is highly associated with heart failure, atrial fibrillation and coronary artery disease, and may be associated with severe heart complications caused by stroke (Wang 2008; Qiang et al. 2013). Compared with non-stroke mice, stroke significantly reduced the expression of serum and cardiac miR-126, and increased miR-126 target genes, vascular cell adhesion protein-1 and monocyte chemotactic protein-1 gene, and cardiac Protein. Therefore, ischemic stroke directly induces cardiac dysfunction, and reducing the expression of miR-126 may lead to cardiac dysfunction after stroke. In addition, circulating miR-145 also increased significantly within 24 h of cerebral ischemia, and the level of circulating miR-145 was positively correlated with the increase in serum inflammatory factor IL-6 (Dharap et al. 2009). MiR-145 has the function of regulating endothelial cells in angiogenesis and vascular stabilization (Climent 2015; Fichtlscherer 2010). Therefore, miRs also play a role in regulating the interaction between the brain and the heart.

In the absence of any clinically evident acute coronary syndrome, CK-MB activity is increased in certain patients with AIS, subarachnoid hemorrhage and head trauma (Norris 1979). Over several years, the activities of AST, CK and CK-MB increased, suggesting that myocardial damage is associated with stroke. The elevation is usually gradual and lasts for several days, unlike in myocardial infarction, where CK-MB peaks and falls within the first 24 h of coronary occlusion (Puleo 1990). Jensen et al. (2007) found that CK-MB was at its highest on the day of stroke symptom onset, and that patients without elevated CK-MB survived significantly longer. The research in this article is consistent with the above research, multifactorial logistic regression showed that CK-MB was an independent risk factor for poor prognosis in non-LAA patients, and for every 1u/L increase in CK-MB, the risk of poor prognosis was 1.126 times higher.

Agnieszka finds that EF is an independent risk factor for neurological prognosis in AIS patients (Rojek 2016). In patients with favorable outcome, the EF was significantly higher compared with patients with poorer prognosis (54.3 ± 7.9 vs. 49.9 ± 9.8, P < 0.005). EF as a useful marker of systolic function is commonly used to guide the application of evidence-based management of patients with heart failure (McMurray 2012). EF is the volume evacuated during ventricular systole as a proportion of the left ventricular volume and is a reliable measure of left ventricular systolic function (McDonagh 1997). Stroke in patients with heart failure is associated with more severe neurological deficits and very high rates of cardiovascular morbidity and mortality (Hays 2006; Vemmos 2012). Haralampos (Milionis 2013) found, stroke-related disability and death rates were higher in low EF patients during follow-up (19.5% vs 7.8% at 1 week, and 36.1% vs 16.5% at 12 months). Consistent with previous reports, patients with low EF exhibited more severe stroke-induced neurological deficits, which may have higher rates of functional impairment and longer hospital stays than EF-preserved patients (Divani et al. 2009; Appelros et al. 2003; Ois 2008). Patients with high EF were reported to have a lower mRS (OR: 0.45, p < 0.001) (Milionis 2013). Similarly, our study also found that patients with low EF had a worse prognosis, with good outcome (mRS ≤ 2) having a higher EF than poor outcome (mRS > 2) (OR: 0.696).

In addition, in our study, the patient’s age (OR: 1.134, 95% CI: 0.963–1.336), NLR (OR: 1.569, 95% CI: 0.925–2.663), albumin (OR: 0.554, 95% CI: 0.255–1.203) and AST (OR: 0.858, 95% CI: 0.684–1.076) significantly affect the prognosis of non-LAA patients. However, by comparing model 2 and model 3, we did not include these factors in the nomogram, which was intended to maintain the ease of use and simplicity of the model. In this article, we found that cardiac parameters are related to the worse prognosis of non-LAA patients, and described the possible pathophysiological processes and molecular mechanisms of their occurrence, which may have some inspiration for the study of brain–heart interaction, and play a certain guiding role in the clinical treatment of this kind of patients.

This article has several shortcomings. The research adopts internal validation but lacks external validation. Internal validation is a common verification method, it is found in George's research most articles use internal validation, and 75% models lack external validation (Siontis et al. 2015). External validation is effective, according to Yvonne’s research, they suggested a minimum of 100 events and 100 nonevents for external validation samples (Vergouwe et al. 2005). According to research, external verification is commonly used in the case of a large sample size (Steyerberg et al. 2003). Therefore, our article adopts an internal validation method. In the following research, we will adopt a multi-center cooperation method for external validation to verify the accuracy of the model. A longer time and larger sample size research will be done later.

## Conclusions

In conclusion, this work suggests that cardiac parameters may be associated with prognosis for non-LAA patients. The nomogram based on cardiac parameters and NIHSS scores established in this paper is a good predictor of the prognosis of patients with non-LAA and helps clinicians to make appropriate treatment decisions.

## Data Availability

The datasets during the current study available from the corresponding author on reasonable request.
